# DMBA acts on cumulus cells to desynchronize nuclear and cytoplasmic maturation of pig oocytes

**DOI:** 10.1038/s41598-017-01870-6

**Published:** 2017-05-10

**Authors:** Zhi-Qiang Song, Xuan Li, Yan-Kui Wang, Zhi-Qiang Du, Cai-Xia Yang

**Affiliations:** 1 College of Animal Science and Technology, Northeast Agricultural University, Harbin, 150030 China; 2 Key Laboratory of Animal Cellular and Genetic Engineering of Heilongjiang Province, Northeast Agriculture University, Harbin, 150030 China

## Abstract

As an environmental pollutant and carcinogen, 7,12-dimethylbenz[a]anthracene (DMBA) can destroy ovarian follicles at all developmental stages in rodents. However, the underlying molecular mechanism remains obscure. In the present study, we aim to address how DMBA affects the *in vitro* maturation and development of porcine oocytes. We discovered that for 20 μM DMBA-treated cumulus-oocyte complexes (COCs), the rate of oocyte germinal vesicle breakdown (GVBD) was significantly altered, and the extrusion rate of first polar body was increased. Moreover, oocytes from 20 μM DMBA-treated COCs had significant down-regulation of H3K9me3 and H3K27me3, up-regulation of H3K36me3, higher incidence of DNA double strand breaks (DSBs) and early apoptosis. In striking contrast, none of these changes happened to 20 μM DMBA-treated cumulus-denuded oocytes (CDOs). Furthermore, 20 μM DMBA treatment increased the reactive oxygen species (ROS) level, decreased mitochondrial membrane potential (Δ Ψm), and inhibited developmental competence for oocytes from both COC and CDO groups. Collectively, our data indicate DMBA could act on cumulus cells via the gap junction to disturb the synchronization of nuclear and ooplasmic maturation, and reduce the developmental competence of oocytes.

## Introduction

As one member of the polycyclic aromatic hydrocarbon (PAH) family, 7,12-dimethylbenz[a]anthracene (DMBA) in the form of persistent organic pollutant exists ubiquitously in the environment. DMBA is mainly produced from incomplete combustion of organic materials, such as gasoline, coal and cigarettes^1^. Due to its toxicity, DMBA can cause not only a wide variety of malicious malignancies^1^, but also human reproductive health issues^2^. Thus, DMBA is registered by the International Agency for Research on Cancer (IARC) as a chemical carcinogen with negative impacts on human health^3^.

Mammalian oocyte originates from the primordial germ cell, undergoes a complex process of meiotic maturation, and then arrests at metaphase II (MII) stage. Only after fertilization or parthenogenetic activation does oocyte start early embryonic development. Meiotic maturation and developmental potency of oocytes are also regulated by the bi-directional communication established by cumulus cells with oocytes through the gap junction^4^. Previous studies demonstrated that cumulus cells could affect gene expression^5^, MAPK activity^6^, postovulatory aging^7^, and reactive oxygen species (ROS) levels of oocytes^8^. *In vitro* cultured oocytes and embryos could often generate ROS, due to lack of proper protection by cumulus cells or *in vivo* milieu^9^, which could damage mitochondria^10^ and cause apoptosis^11^.

Following germinal vesicle breakdown (GVBD) during first meiosis, direct exposure of chromosomes in the ooplasm opens up a very sensitive time window, when chemicals^12, 13^ (e.g. toxins or drugs) can cause the de-synchronization of nuclear and cytoplasmic maturation, and affect developmental potency. Exposure to chemicals can dramatically induce excessive production of ROS^13^, which can cause poor embryo quality, delayed and even arrested embryo development^14, 15^. Chemicals can also induce DNA double strand breaks (DSBs) in oocytes^16^. When DSBs occur, histone H2A.X is phosphorylated at serine 139 to γH2A.X, and forms foci at the DSB sites^17^. Mouse oocytes with DSBs undergo apoptosis *in vivo* ^17^, whereas *in vitro*, they can complete polar body extrusion once entering GVBD stage and form multiple pronuclei or numerous micronuclei after parthenogenetic activation^16^. In addition, chemicals can change dynamically the epigenetic methylation status of lysine residues on histone 3, which is critical to preimplantation development of embryos^18, 19^.

DMBA can impose toxicity on ovary, and destroy follicles at all developmental stages in a dose-dependent manner, partially through the alteration of gap junction, DNA damage, and gene expression in rodents^20–23^. In pigs, there is only one report that DMBA can significantly increase the GVBD rate for *in vitro* cultured COCs in medium supplemented with hypoxanthine, but without hormones and EGF^24^. Nevertheless, the molecular mechanism how DMBA exerts its effect on oocyte meiotic maturation and developmental ability remains obscure, especially in pigs. More close to humans in size, physiology and genetics than rodents, pig is considered as a better animal model^25^. In the current study, we used the *in vitro* maturation system of pig COCs and CDOs, to investigate DMBA’s effects on nuclear and ooplasmic maturation of oocytes, and subsequent embryo development, with regard to DSBs, apoptosis, histone methylation modification, ROS and mitochondrial membrane potential (Δ Ψm).

## Results

### DMBA alters oocyte meiotic cycle by changing p-ERK1/2 protein level

**Figure 1:**
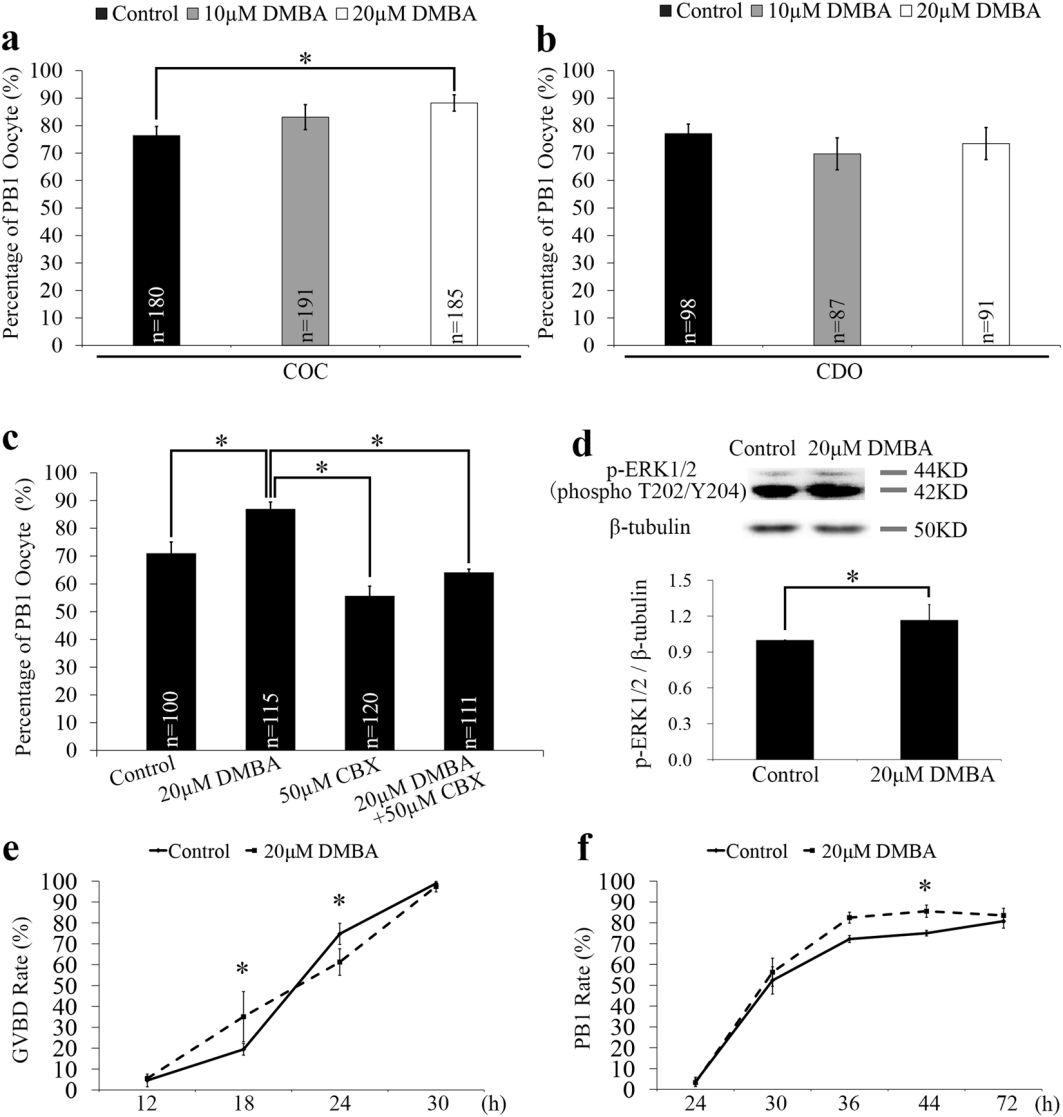
Effects of DMBA exposure on PB1 extrusion, p-ERK1/2 level and meiotic cycle of oocytes. (**a**) The PB1 rates of oocytes from COC and CDO groups treated with DMBA for 44 h. (**b**) The PB1 rates of oocytes from CDO groups treated with DMBA for 44 h. (**c**) The PB1 rates of oocytes from COCs treated with DMBA and/or CBX for 44 h. (**d**) The lysates of 200 oocytes collected at 44 h from control and DMBA treated COCs were subjected to western blot analysis and bands of p-ERK1/2 and β-tubulin were cropped out of blot to display (full-length blot as shown in Supplementary Fig. S3). The relative protein level of p-ERK1/2 was quantified using Image J software. (**e**) The GVBD rates of oocytes from COCs collected at 12 h, 18 h, 24 h and 30 h. (**f**) The PB1 rates of oocytes from COCs collected at 30 h, 36 h, 44 h and 72 h. *Indicates significant differences at P < 0.05 level between groups.

In porcine COCs maturated *in vitro* (IVM), we observed that DMBA exposure suppressed the expansion of cumulus cells at 24 h, and promoted the detachment of cumulus cells from the oocyte cargo at 44 h, in both 10 μM and 20 μM DMBA treatment groups (Supplementary Fig. S1). The rate of first polar body (PB1) extrusion at 44 h was significantly higher in 20 μM DMBA group than the control group (88.2% vs. 76.4%; P < 0.05; Fig. 1a). However, for the CDO IVM system, no significant differences existed between the DMBA (69.7% for 10 μM and 73.5% for 20 μM) and the control (77.1%) groups (P > 0.05; Fig. 1b). As a result, we chose 20 μM DMBA to carry out the remaining experiments.

To examine whether cumulus cells mediated the DMBA induced rise of oocyte PB1 rate in the COC system, we used carbenoxolone (CBX), an inhibitor of gap junction, to block the bi-directional communication between cumulus cells and oocyte. The oocyte PB1 rate in the 20 μM DMBA + 50 μM CBX group dropped significantly when compared to the 20 μM DMBA group (64.1% vs. 87.0%; P < 0.05), but showed no significant difference when compared to the 50 μM CBX only group (64.1% vs. 55.7%) (P > 0.05; Fig. 1c). Moreover, for CDOs, treatment by 50 μM CBX did not trigger significant changes of the PB1 rate, as compared to the control group (68.3% vs. 71.0%; P > 0.05; Supplementary Fig. S2).

Additionally, in COCs, DMBA treatment significantly increased level of activated MAPK (p-ERK1/2) in oocytes as compared to the control group (P < 0.05; Fig. 1d; Supplementary Fig. S3), which indicates that DMBA treatment could act on cumulus cells to activate the MAPK pathway within oocytes via the gap junction and thus to increase the PB1 rate of oocytes.

In order to clarify DMBA’s effect on oocyte nuclear maturation events in COCs, we further classified the nuclear stages of oocytes at different time points during IVM (Supplementary Fig. S4). DMBA treatment increased significantly the GVBD rate at 18 h, but decreased significantly at 24 h, respectively (P < 0.05; Fig. 1e). Following GVBD, compared to the control group, DMBA treatment gradually increased the PB1 rate, significantly at 44 h (85.6% vs. 75.0%; P < 0.05), but not at 72 h (83.5% vs. 80.8%; P > 0.05; Fig. 1f).

### DMBA reduces the developmental competence of mature oocytes

**Figure 2:**
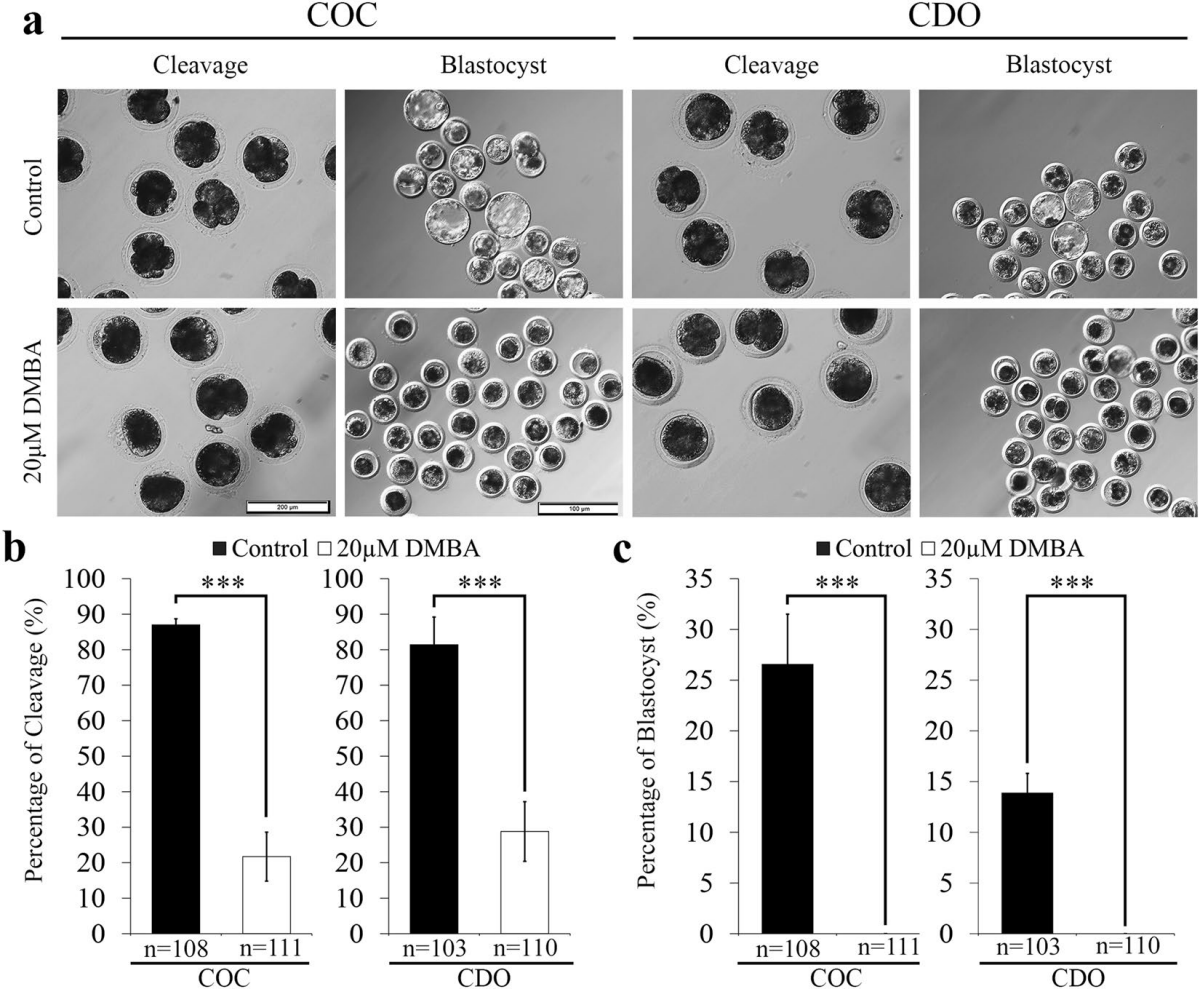
DMBA treatment during IVM affected developmental ability of mature oocytes. (**a**) Morphology of cleaved parthenotes (Scale bar, 200 μm) and blastocysts (Scale bar, 500 μm). (**b**) Percentages of cleaved parthenotes. (**c**) Percentages of parthenots developed to blastocysts. ***P < 0.001.

In the present study, parthenogenetic activation was used to evaluate the developmental competence of oocytes^26^, rather than *in vitro* fertilization, considering the exclusion of confounding effects from sperm that could affect the developmental potential of embryos^27^. In COCs, 20 μM DMBA significantly decreased the cleavage rate of parthenotes in comparison to the control group (21.7% vs. 86.2%; P < 0.001), and all parthenotes failed to develop to blastocyst (Fig. 2). In CDOs, we found similar effects that cleavage rate of parthenotes was significantly decreased (28.8% vs. 81.4% of the control; P < 0.001) and no blastocyst was obtained from 20 μM DMBA treated oocytes (Fig. 2). After staining the developmentally arrested 1-cell parthenotes derived from DMBA treated oocytes, we found that 100% of them were activated, and different numbers of pronuclei (from 1 to 6) were formed (Supplementary Fig. S5). These results indicated that DMBA treatment impaired the developmental potency of MII oocytes in both COC and CDO systems.

### DMBA induces DSBs and apoptosis in mature oocytes via cumulus cells

**Figure 3:**
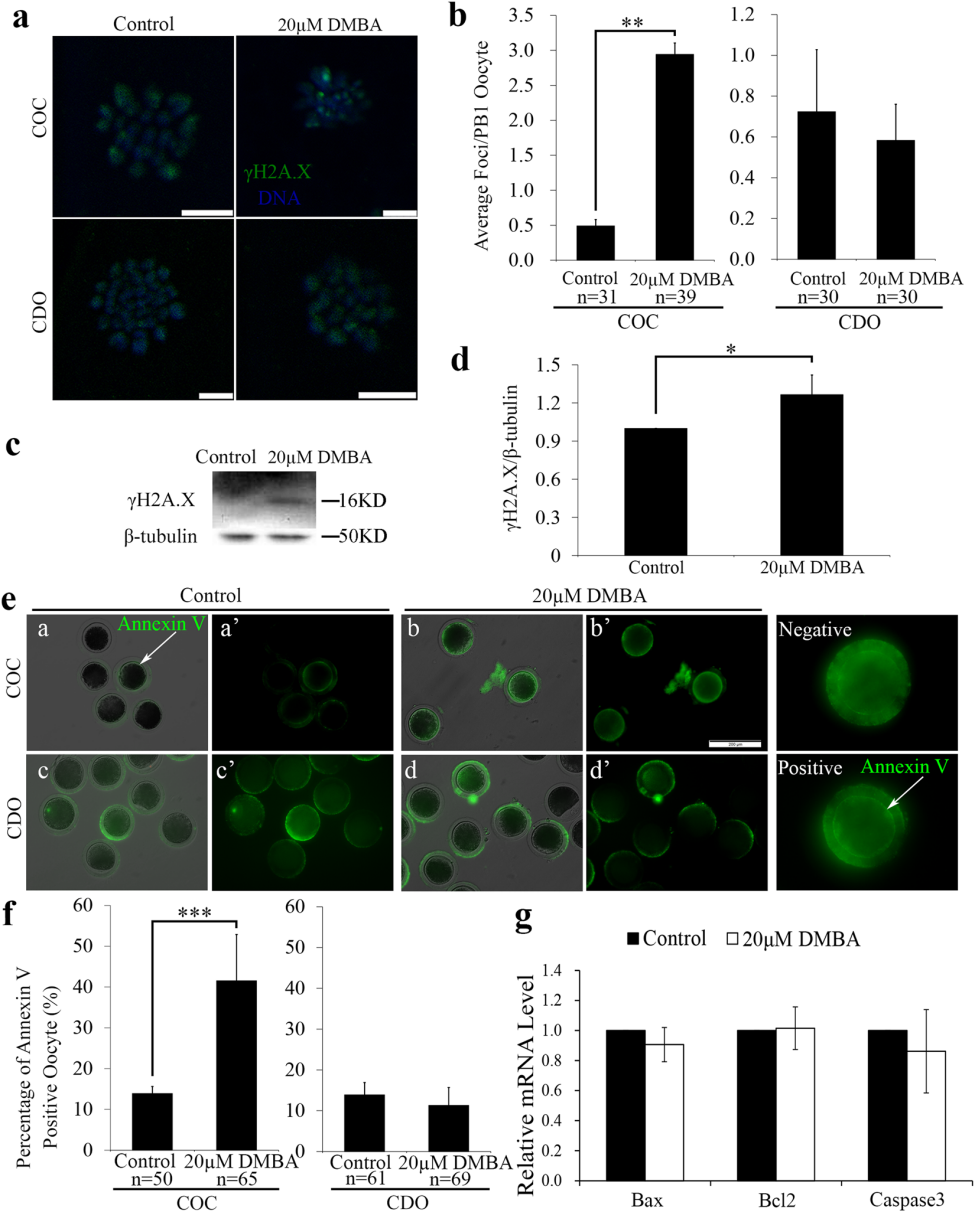
DMBA induced DNA damage and apoptosis in mature oocytes from COCs. (**a**) Representative images of γH2A.X immunostaining. Scale bar, 500 μm. (**b**) Average number of DSB foci per mature oocyte. (**c**) The lysates of 200 oocytes collected at 44 h from control and DMBA treated COCs were subjected to western blot analysis and bands of γH2A.X and β-tubulin were cropped out of blot to display (full-length blot as shown in Supplementary Fig. S3). (**d**) Relative quantification of γH2A.X protein level. (**e**) Representative images of Annexin-V staining (green) in mature oocytes. Scale bar, 200 μm. (**f**) Percentages of apoptosis positive oocytes. (**g**) The relative mRNA level of Bax, Bcl2 and Caspase3 in mature oocytes. *P < 0.05; **P < 0.01 and ***P < 0.001.

We further checked if DMBA treatment would cause DNA damage and induce the early apoptosis in mature oocytes. γH2A.X staining was used as a marker to label the sites of DNA DSBs, and western blots were used to check γH2A.X protein levels. In COCs, 20 μM DMBA treatment significantly increased number of DSB foci (2.9 vs. 0.5; P < 0.01; Fig. 3a,b) and γH2A.X protein levels in oocytes (P < 0.05; Fig. 3c,d) as compared to that in the control group, suggesting DMBA induced oocyte DNA damage. In CDOs, no difference of DSB foci number was found between 20 μM DMBA treated and the control groups (0.6 vs. 0.7; P > 0.05; Fig. 3a,b).

Annexin-V staining was performed to assess early apoptosis in mature oocytes. In COCs, significantly higher percentage of apoptotic oocytes was revealed in the 20 μM DMBA group than that in the control group (41.5% vs. 13.9%; P < 0.001; Fig. 3e,f). Further assays on the relative mRNA expression levels showed no differences for three apoptosis-related genes (*Bax*, *Bcl2* and *Caspase3*) in the 20 μM DMBA treated COC group when compared to the control groups (P > 0.05; Fig. 3g). In CDOs, regarding percentage of oocyte apoptosis, we did not find any difference between the control and 20 μM DMBA groups (13.0% vs. 11.0%; P > 0.05; Fig. 3e,f). Together, these results indicated that DMBA induced DSBs and apoptosis in mature oocytes via cumulus cells.

### DMBA acts on cumulus cells to modify histone methylation in oocytes

**Figure 4:**
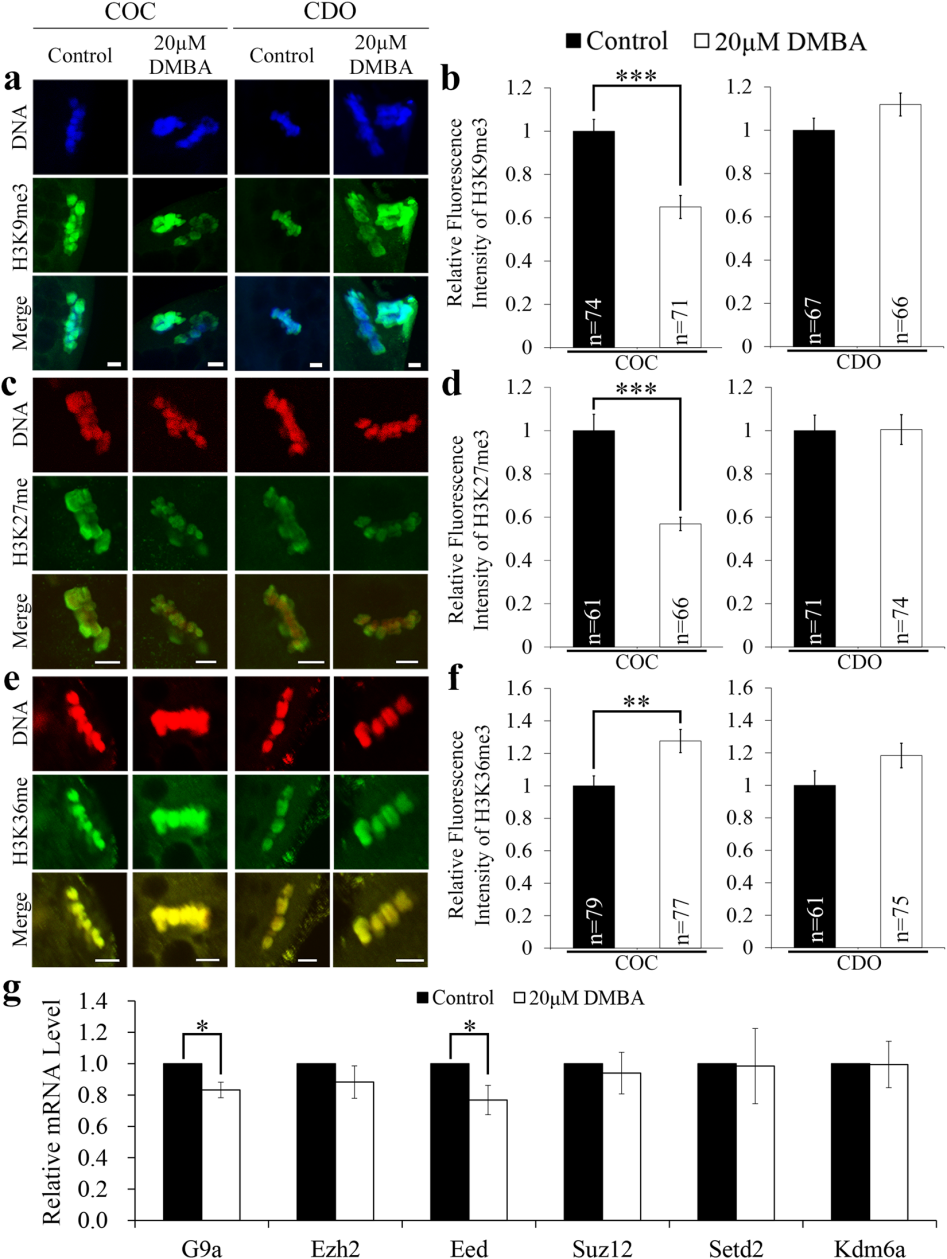
DMBA modified histone methylation markers of mature oocytes from COCs. Representative fluorescence images of mature oocytes were presented for H3K9me3 (**a**), H3K27me3 (**c**) and H3K36me3 (**e**). Scale bar, 5 μm. The relative fluorescence intensity of H3K9me3 (**b**), H3K27me3 (**d**) and H3K36me3 (**f**) were presented as mean ± SEM. (**g**) The relative mRNA level of G9a, Ezh2, Eed, Suz12, Setd2 and Kdm6a in mature oocytes. *P < 0.05; **P < 0.01 and ***P < 0.001.

We further compared the tri-methylation levels of histone H3 at three lysine positions in mature oocytes, 9 and 27 (H3K9me3, H3K27me3), and 36 (H3K36me3), indicating inactive and active chromatin status, as reported previously^18, 28^. In COCs, exposure to 20 μM DMBA significantly decreased H3K9me3 and H3K27me3 levels (P < 0.001; Fig. 4a–d), significantly increased the H3K36me3 level (P < 0.01; Fig. 4e,f). In the CDO system, 20 μM DMBA treatment did not significantly change levels of all these three markers (P > 0.05; Fig. 4a–f). These data suggested that DMBA could directly act on cumulus cells to affect the epigenetic status of histones in oocytes.

Moreover, we checked the mRNA expression levels of six genes involved in the establishment of epigenetic modifications on these three histone methylation markers (*G9a*, *Ezh2*, *Eed*, *Suz12*, *Setd2*, *Kdm6a*). *G9a* and *Eed* were found to be significantly down-regulated in mature oocytes from DMBA-treated COC group when compared to the control group (P < 0.05; Fig. 4g).

### DMBA increases ROS production and decreases mitochondrial Δ Ψm in oocytes

**Figure 5:**
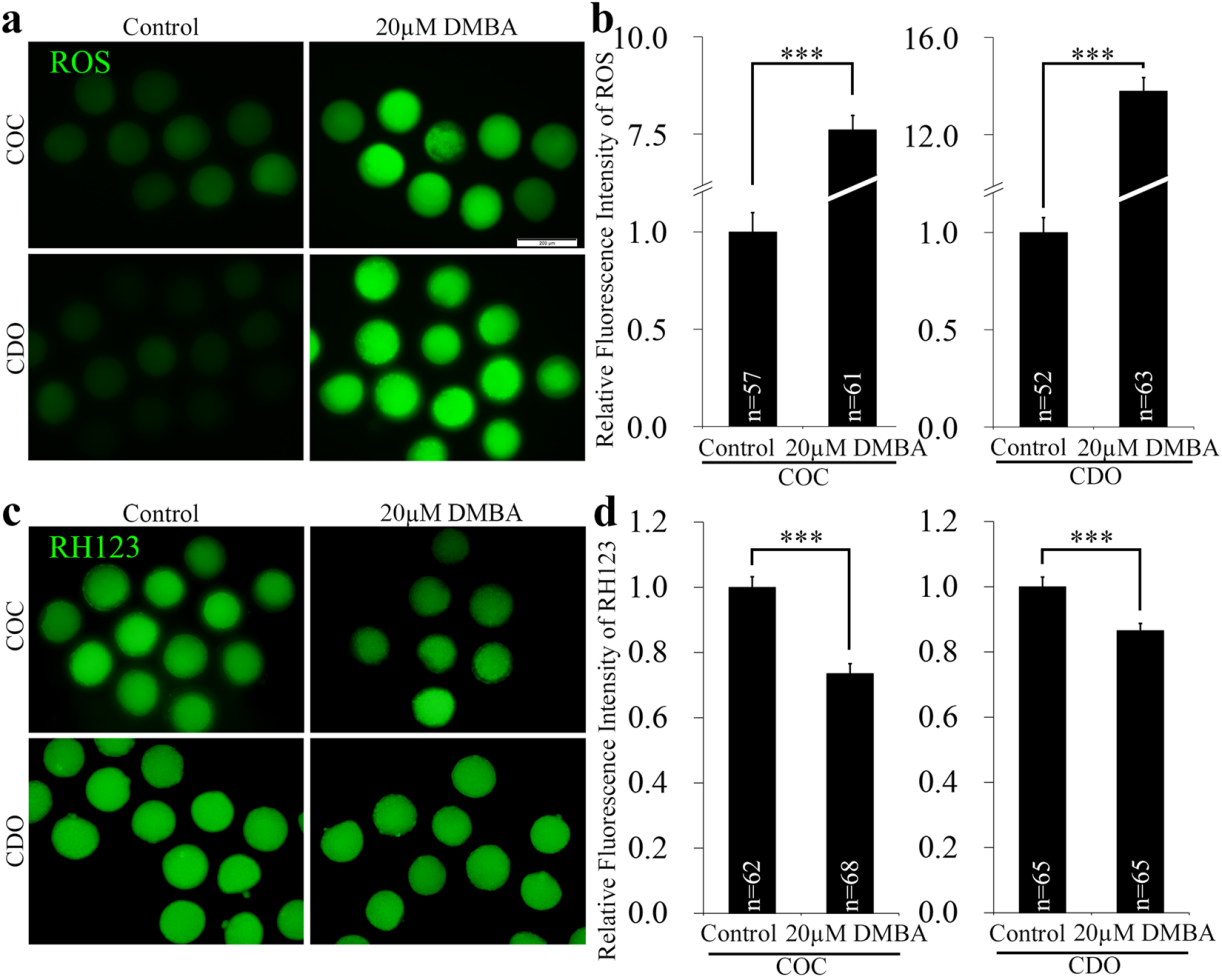
DMBA increased ROS level and decreased mitochondrial membrane potential in porcine mature oocytes. Representative fluorescence images were taken from mature oocytes stained with DCFH-DA (**a**) and RH123 (**c**). Scale bar, 200 μm. The graphs were presented as relative fluorescence intensity of ROS (**b**) and RH123 (**d**) in mature oocytes. ***P < 0.001.

Cytoplasmic ROS level and mitochondrial Δ Ψm correlate with the cytoplasmic quality of oocytes, which determines the subsequent developmental competency of oocytes and embryos. In the COC system, exposure to 20 μM DMBA significantly elevated the ROS level (7.6 vs. 1.0; P < 0.001) and decreased mitochondrial Δ Ψm (0.7 vs. 1.0; P < 0.001), as compared to the controls (Fig. 5). In CDOs, 20 μM DMBA treatment significantly boosted the ROS level (13.8 vs. 1.0; P < 0.001) and dramatically decreased mitochondrial Δ Ψm (0.9 vs. 1.0; P < 0.001), when compared to the control groups (Fig. 5). Taken together, DMBA could degrade the cytoplasmic quality of oocytes for both COCs and CDOs.

## Discussion

Global industrial development, especially in developing countries, generates many of chemical wastes, including DMBA. DMBA poses great threats and concerns over not only environmental standards, but also human health issues. In reproductive biology, DMBA has been found to be toxic on ovarian and follicular development in rodents^20–23^. In the current study, we further investigated DMBA’s effects on porcine oocytes with and without cumulus cells enclosed, by assessing meiotic progression, developmental potency, DSB, early apoptosis, histone methylation, oxidative stress and mitochondrial function. Our results indicated that DMBA could have detrimental effects on nuclear and ooplasmic maturation of porcine oocytes through multiple pathways, and cumulus cell is an important mediator to some of DMBA’s effects.

DMBA treatment can increase the extrusion rate of PB1 for oocytes enclosed with cumulus cells, but without effect on CDOs. Blocking gap junction by CBX inhibited DMBA’s effect on elevating the PB1 rate of COCs. These data indicated that cumulus cells act through gap junction to regulate DMBA’s effect on oocyte nuclear maturation. Previously, it was found that mutagenic metabolites from DMBA generated by porcine ovarian granulosa cells could migrate into surrounding cells, and cause mutations there^29^. To understand the underlying mechanism, we examined p-ERK1/2 level in porcine oocytes from COCs, and found DMBA incubation increased p-ERK1/2 protein level. MAPK cascade is one of the principal signal transduction pathway during meiotic progression, with important roles on meiotic resumption, spindle assembly, MII arrest and pronuclear formation^30^. Thus, one reason that DMBA could induce the increase of oocyte PB1 rate is through increasing p-ERK level. In addition, we also found DMBA could increase the GVBD rate at 18 h of IVM, but decreased at 24 h, suggesting nuclear event of meiotic resumption was altered by DMBA. One previous study used a different kind of culture medium, with hypoxanthine but without hormones and EGF^24^, and found that porcine COCs treated by 10 µM DMBA for 24 h had significantly increased GVBD rate. Thus, differences of the culture system could influence DMBA’s effects on meiotic resumption. In summary, we showed that DMBA acts on cumulus cells to influence porcine oocyte nuclear maturation. However, the underlying molecular mechanism remains to be determined.

We observed that most parthenotes arrested at 1-cell stage, and the formation of 1–6 pronuclei, after treating both COCs and CDOs by DMBA. These results indicated that although DMBA treated MII oocytes could complete the transition from metaphase to interphase, most parthenotes were blocked at 1-cell. Multiple reasons could help to explain this phenomenon. Firstly, we found already that DMBA treatment can cause the higher p-ERK level, and high MAPK activity in mouse oocytes prior to activation is essential for pronuclear formation^31^ and the fusion of nucleolus precursor bodies after activation^32^. Secondly, DNA DSBs in mouse mature oocytes after parthenogenetic activation were reported to induce the formation of multiple pronuclei or numerous micronuclei^16^. Our results also showed that multiple pronuclei formed in 1-cell arrest parthenotes derived from DMBA treated mature oocytes, where DSBs also occurred. However, DSBs appeared only in oocytes from COCs treated by DMBA, but not in CDOs. This could be explained by the fact that cumulus cells were previously shown to control the DSB response^33^ and the DSB-induced formation of nuclear actin filament in oocytes^34^. Thirdly, apoptosis could be induced by DSBs in oocytes^17^, and we found DMBA could induce higher incidence of early apoptosis, but again, only in the COC system. Therefore, our data suggested that surrounding cumulus cells could regulate DSB and its downstream events within oocytes treated by DMBA. Since both DNA damage and early apoptosis in oocytes affected following embryo development^16, 35^, these might be reasons also for the developmental failure in COCs treated by DMBA.

Studies showed that chemicals/drugs could affect levels of histone methylation markers at different lysine positions of histone H3 in meiotic mouse^36, 37^ and pig^18^ oocytes. In mice, H3K9me3 is significantly higher in the maternal pronuclei, while H3K27me3 and H3k36me3 are found exclusively in the maternal pronuclei of fertilized embryos. These epigenetic histone markers play critical roles in the regulation of embryonic genome activation, maternal *Xist* activation, ICM (inner cell mass)/TE (trophectoderm) lineage specification and preimplantation development^19^. Our results showed the increased level of H3K36me3 and the decreased levels of H3K9me3 and H3K27me3 in mature oocytes only in the COC system, indicating that cumulus cells control DMBA induced alternation of oocyte histone modifications. And, the epigenetic changes triggered by DMBA are more likely to be passed into maternal pronuclei to interrupt embryo development. Furthermore, *G9a* and *Eed* related to histone modification significantly decreased their transcript abundances after DMBA treatment in COCs, which could be due to elevated degradation by DMBA treatment, since MII oocytes are transcriptionally inactive.

ROS also play a significant role in oocyte maturation and embryo development^38^. Oxidative stress occurs when excessive ROS appears under chemical treatment^39^, which could then damage embryo development, but does not affect nuclear maturation for pig oocytes^40^. Our results showed that DMBA treatment resulted in excessive ROS generation in pig oocytes in both COC and CDO systems. Moreover, even higher ROS level was found in CDOs, which might result from the decreased antioxidant defense due to the loss of cumulus cells^8^. It has been shown that sustained exposure to ROS induced mitochondria damage^10^. Therefore, we examined mitochondrial Δ Ψm level and found DMBA exposure lowered mitochondrial Δ Ψm levels in both COC and CDO systems, indicating mitochondrial function was disturbed in oocytes exposed to DMBA. Both oxidative stress and mitochondrial dysfunction have been reported to link with developmental block of embryos cultured *in vitro* ^9, 41^. Thus, DMBA induced oxidative stress and mitochondrial dysfunction were important reasons for the failure of embryo development.

Take together, DMBA triggers changes of meiotic cycle, DSB, early apoptosis and histone methylation modification only in oocytes enclosed with cumulus cells, and induces sharp ROS rise and mitochondrial Δ Ψm decrease in oocytes no matter whether cumulus cells are present or not. Cumulus cells play an important role in mediating DMBA’s effects on nuclear and ooplasmic maturation, which can reduce oocyte developmental capacity for both COC and CDO systems.

## Methods

### Ethics statement and chemicals

All materials collected and experimental procedures taken in this study were reviewed and approved by the Animal Care Commission and Ethics Committee of Northeast Agricultural University P. R. China. The methods were carried out in accordance with the approved guidelines. Chemicals and reagents used were purchased from Sigma Chemical Company (St. Louis, MO, USA), unless mentioned specifically.

### Oocyte *in vitro* maturation (IVM)

Porcine oocytes were collected, washed and *in vitro* matured as previously described^42^. Briefly, porcine ovaries were collected from gilts at a local slaughterhouse, and transported to the laboratory in a thermos bottle containing saline solution with 100 IU/ml penicillin and 0.05 mg/ml streptomycin within 3 h (30–35 °C). The follicular fluids from 3–6 mm antral follicles were aspirated using an 18-gauge needle attached to a 10 ml disposable syringe. After washing three times with TL-HEPES-PVA, COCs with multiple layers of intact cumulus cells and uniform ooplasm were selected for further experiments. To prepare CDOs, the cumulus cells of COCs were separated from oocytes by gentle vortexing in 0.1% hyaluronidase in TL-HEPES-PVA. The selected COCs and denuded CDOs were transferred into 500 µl of maturation medium (TCM 199 medium (Gibco BRL, Grand Island, NY) supplemented with 0.1% PVA, 3.05 mM D-glucose, 0.91 mM sodium pyruvate, 1 µg/ml gentamicin, 0.57 mM cysteine, 0.5 µg/ml luteinizing hormone (LH), 0.5 µg/ml follicle stimulating hormone (FSH), 10ng/ml epidermal growth factor (EGF)) covered with mineral oil in a 24-well plate (Nunc, Roskilde, Denmark), and matured for 44 h in an incubator (Thermo, USA) (39 °C, 5% CO_2_ and saturated humidity).

### DMBA and CBX treatment

DMBA (D3254, Sigma) was dissolved in dimethyl sulfoxide (DMSO, D2650, Sigma) to make 20 mM stock solution, and then added into the oocyte maturation medium to obtain the final concentrations of 10 μM and 20 μM, with DMSO concentration less than 0.1%. Control maturation medium had the same concentration of DMSO as DMBA groups. We searched the literature about DMBA concentration used in *in vitro* experiments, and summarized the results in the Supplementary Table S1. The 10 μM DMBA concentration was initially selected based on a previous study on pig COCs using culture medium supplemented with hypoxanthine but without EGF, FSH and LH^24^ (component differences of culture medium were summarized in the Supplementary Table S2). Because 10 μM DMBA in our culture system did not reach statistical significance for PB1 rate (Fig. 1a), we then used 20 μM DMBA in our experiments, which caused significant changes as reported here (Fig. 1a). Carbenoxolone disodium salt (CBX, C4790, Sigma) was dissolved in water, and then added into the oocyte maturation medium to obtain the final concentrations of 50 μM^43^.

### Assessment of oocyte nuclear status

Status of oocyte nuclei was evaluated by Hoechst33342 staining^42^. Briefly, cumulus cells were stripped off and oocytes were fixed in 4% (w/v) paraformaldehyde for 40 min at room temperature (RT), stained with 10 µg/ml Hoechst33342 in the phosphate buffered saline (PBS) solution for 10 min, and then mounted onto slides for examination under an inverted fluorescence microscope (Olympus, Tokyo, Japan). The nuclear status was classified as germinal vesicle (GV), pre-metaphase I (pre-MI), metaphase I (MI), anaphase I and telophase I (AI + TI) and metaphase II (MII).

### Measurement of reactive oxygen species (ROS) and mitochondrial membrane potential (Δ Ψm)

Intracellular ROS and mitochondrial Δ Ψm levels of MII stage oocytes were measured by the 2′,7′-dichlorofluorescein (DCF) fluorescence assay and Rhodamine 123 (RH123) staining as described previously^42^. Briefly, oocytes were incubated in 10 µM 2′,7′-dichlorodihydrofluorescein diacetate (DCFH-DA) or 5 μg/ml RH123 in PBS for 30 min in dark, followed by three washes with PBS, and then put into 10 µl droplets of PBS to take the fluorescence images under the inverted fluorescence microscope (Olympus, Tokyo, Japan). All images were taken precisely at the same parameters for all groups. The fluorescence intensity was quantified using the ImageJ software^44^.

### Assessment of oocytes with apoptosis

Oocytes with PB1 collected at 44 h were examined by the Annexin V-FITC Apoptosis Detection Kit (Sigma, USA) according to the manufacturer’s instruction and previous report^45^. Briefly, oocytes were washed three times with binding buffer and then incubated with Annexin V-FITC solution (1:100 in binding buffer) at room temperature for 20 min in the dark. After three washes with binding buffer, samples were mounted on glass slides and examined under an epifluorescence microscope (Olympus, Tokyo, Japan) equipped with a digital camera. Oocytes with Annexin-V staining signal full of the plasma membrane were classified as apoptosis positive oocytes whereas oocytes without signal or with partial signal on plasma membrane were classified as apoptosis negative ones.

### Immunocytochemistry

For histone methylation staining, MII oocytes were fixed with 4% (w/v) paraformaldehyde in PBS for 40 min at RT, and permeabilized with 1% Triton-X100 in PBS at 4 °C overnight. On the next morning, oocytes were blocked in 1% bovine serum albumin (BSA) in PBS for 1 h at RT followed by incubation in rabbit anti-H3K9me3, anti-H3K27me3 and anti-H3K36me3 antibodies (1:50 in the blocking buffer, Abclonal, Nanjing, China) overnight at 4 °C. On the following day, after three washes in PBS supplemented with 0.1% Triton X-100 and 0.01% Tween-20 (PBST, 10 min each wash), oocytes were incubated in second antibody (FITC conjugated goat anti-rabbit IgG(H + L)) for 1 h at RT. Then washed three times by PBST and samples were stained with Hoechst33342 (10 µg/ml in PBS) or propidium iodide (10 µg/ml in PBS) for 10 min at RT. Finally, oocytes were mounted onto glass slides in slow-fade gold antifade reagent (Life Technologies, USA). For γH2A.X immunostaining, some modifications were made as described below. Oocytes were permibilized in 1% Triton-X100 at RT for 30 min, blocked using 3% BSA, incubated in mouse anti-γH2A.X antibody (1:1500, Abcam, Cambridge, UK) overnight at 4 °C and FITC conjugated goat anti-mouse IgG(H + L) for 1 h. Negative controls were incubated without the primary or secondary antibody. A fluorescence microscope (Nikon, Japan) was used to take fluorescence digital images and fluorescence intensity was quantified by determining the median pixel intensity using the ImageJ software. Representative images were taken using a laser-scanning confocal microscope (Leica, Germany).

### Parthenogenetic activation

Cumulus-denuded MII pig oocytes were parthenogenetically activated and parthenotes were observed and recorded according to the method described previously^42^. Briefly, oocytes were equilibrated, stimulated by applying three pulses of 1.2 kV/cm direct current for 20 μsec using Electrocell Manipulator (BTX830, USA) and then transferred into PZM-3 containing 2.5 mM 6-(dimethylamino)purine and 5 μg/mL cytochalasin B to culture for 4 h in the incubator (39 °C, 5% CO_2_, saturated humidity). After activation, parthenotes were cultured in PZM-3 medium covered with mineral oil in the incubator (5% CO_2_ in humidified air at 39 °C) for seven days. Embryonic cleavage and blastocyst rates were evaluated at 48 h and 168 h post-activation (hpa), respectively. Parthenotes collected at day 7 were stained using Hoechst33342 to assess the nuclear status.

### Real-time quantitative PCR

Total RNA from 60 MII oocytes was extracted by the RNeasy Mini Kit (Qiagen, Germany), combined with the RNase-free DNase (Qiagen) treatment to remove the genomic DNA. First strand cDNA was synthesized in a 20 μl reaction volume using the ABI kit (Life technologies). Primers were designed by the Primer-blast and shown in Supplementary Table S3. 10 µl PCR reaction volume, including 1 µl cDNA template, primers and Roche Fast Start Universal SYBR green master mix (Roche Molecular Systems), was used to quantify transcripts on a 7500 real-time PCR detection system (Applied Biosystems, Carlsbad, CA, USA). PCR parameters were set up as follows: 95 °C for 10 min, followed by 40 cycles at 95 °C for 15 sec and 60 °C for 1 min. Each sample was tested in triplicate and Ywhag was used as the reference gene. Relative abundance of transcripts was calculated using the comparative Ct (2^−ΔΔCt^) method.

### Western blot

A total of 200 live oocytes devoid of cumulus cells at 44 h were lysed in 2 × Laemmli sample buffer (SDS sample buffer with 2-mercaptoethanol, 2 mM PMSF, 2 mM Phosphatase Inhibitor Cocktail), and boiled at 100 °C for 4 min. Proteins were separated by SDS polyacrylamide gel electrophoresis (PAGE) and transferred onto the nitrocellulose membrane (Transgen Biotech, Beijing, China). The membrane was blocked for 1 h at RT in 5% BSA in PBS. To detect both p-ERK1/2 (phospho T202/Y204) and γH2A.X (phospho S139), blot was cut into two parts containing the proteins above and below the 25 kDa marker and incubated separately overnight at 4 °C in 0.5% BSA with 1:1000 polyclonal rabbit anti-p-ERK1/2 antibody (Abclonal, Nanjing, China) for the upper part of the membrane and with 1:1000 monoclonal mouse anti-γH2A.X antibody (Abcam, Cambridge, UK) for the lower part. The next day, membrane was washed three times in PBST and incubated for 1 h with anti-rabbit or anti-mouse HRP-conjugated secondary antibodies (1:10000 in 0.5% BSA). Three washes later, the membrane was processed using enhanced chemiluminescence (ECL) detection system (Shenzhen, China). For reprobing, the membrane was washed in stripping buffer (Beyotime Biotechnology, Shanghai, China) at RT for 7 min to strip off bound antibody after ECL detection. The membrane was re-probed with 1:1000 polyclonal mouse anti-β-tubulin antibody (Transgen Biotech, Beijing, China) using the above described procedure. Three replicates were performed for all experiments.

### Statistical analysis

At least three biological replicates were performed for each experiment with results expressed as mean ± SEM. Comparison between two groups was performed using Student’s t test (JMP 11.0, SAS Institute Inc., Cary, NC, USA). All the percentage data were analyzed using the Chi-square test, followed by multiple comparisons with Bonferroni correction (SPSS 19.0).

## Electronic supplementary material

Supplementary information
