# Supplementary material for: DMBA acts on cumulus cells to desynchronize nuclear and cytoplasmic maturation of pig oocytes
Source: Sci Rep. 2017 May 10;7:1687. doi: 10.1038/s41598-017-01870-6 (PMC5431913; doi:10.1038/s41598-017-01870-6)

DMBA acts on cumulus cells to desynchronize nuclear and cytoplasmic maturation of pig oocytes

Zhi-Qiang Song[1], Xuan Li[1], Yan-Kui Wang[1], Zhi-Qiang Du[1,2*], Cai-Xia Yang[1,2*]

Supplementary information

Supplementary Table S1：The information of DMBA concentration used in vitro in previous reports.

Level of material	Species and material type	DMBA concentration	Culture/ treatment time	Reference	Note
Somatic cell	Human granulosa cell	5μM	12h	Bengtsson et al., 1988	+HCG
	Human T cell	1,3, 10, 30μM	100sec	Archuleta et al., 1993	+10% FBS
	Mice thymus cell	3,10,30μM	4h	Burchiel et al., 1992	+10% FBS
	Mice langerhans cell	32μM, 64μM	24h	Modi et al., 2012	+10% FBS
	Pig ovarian granulosa cell	5μM	48h	Becedas et al., 1993	+10% FCS
Ovary	Mice ovary	50nM	4days	Sobinoff et al., 2011	+5% FCS
	Mice ovary	1μM	72h,6days	Matikainen et al., 2001	+3mg/ml BSA
	Neonatal rat ovary	12.5nM,75nM	1,2,4,8days	Madden et al., 2014	+1mg/ml BSA +1mg/ml Albumax
Germ cell	Mytilus sperm	0,5,10,15,20μM	10min	Togo and Morisawa, 2004	Seawater only
	Ascidian oocyte	10, 20μM	1h	Lambert., 2005, 2011; Robert et al., 1999	Seawater only
	Pig COC	0.01,0.1,1,10μM	24h	Su et al., 1999	+hypoxanthine - hormones

Abbreviation in Supplementary Table S1

COC:cumulus -oocyte complexe; HCG:human chorionic gonadotropin; FCS:fetal calf serum; FBS:fetal bovine serum; BSA: bovine serum albumin.

References (cited in Supplementary Table S1)

1. Bengtsson, M., Hamberger, L. & Rydström, J. Metabolism of 7,12-dimethylbenz(a)anthracene by different types of cells in the human ovary. *Xenobiotica.* **18,** 1255-1270 (1988).

2. Archuleta, M. M., Schieven, G. L., Ledbetter, J. A., Deanin, G. G. & Burchiel, S. W. 7,12-Dimethylbenz[a]anthracene activates protein-tyrosine kinases Fyn and Lck in the HPB-ALL human T-cell line and increases tyrosine phosphorylation of phospholipase C-gamma 1, formation of inositol 1,4,5-trisphosphate, and mobilization of intracellular calcium. *Proc Natl Acad Sci USA.* **90,** 6105-6109 (1993).

3. Burchiel, S. W. et al. DMBA-induced cytotoxicity in lymphoid and nonlymphoid organs of B6C3F1 mice: relation of cell death to target cell intracellular calcium and DNA damage. *Toxicol Appl Pharmacol.* **113,** 126-132 (1992).

4. Modi, B. G. et al. Langerhans cells facilitate epithelial DNA damage and squamous cell carcinoma. *Science.* **335,** 104-108 (2012).

5. Becedas, L. et al. Metabolism of polycyclic aromatic hydrocarbons to mutagenic species by rat and porcine ovarian granulosa cells: detection by cocultivation with V79 Chinese hamster cells. *Reprod Toxicol.* **7,** 219-224 (1993).

6. Sobinoff, A. P., Mahony, M., Nixon, B., Roman, S. D. & McLaughlin, E. A. Understanding the Villain: DMBA-induced preantral ovotoxicity involves selective follicular destruction and primordial follicle activation through PI3K/Akt and mTOR signaling. *Toxicol Sci.* **123,** 563-575 (2011).

7. Matikainen, T. et al. Aromatic hydrocarbon receptor-driven Bax gene expression is required for premature ovarian failure caused by biohazardous environmental chemicals. *Nat Genet.* **28,** 355-360 (2001).

8. Madden, J. A., Hoyer, P. B., Devine, P. J & Keating, A. F. Acute 7,12-dimethylbenz[a]anthracene exposure causes differential concentration-dependent follicle depletion and gene expression in neonatal rat ovaries. *Toxicol Appl Pharmacol.* **76,** 179-187 (2014).

9. Togo, T. & Morisawa, M. GPI-anchored aminopeptidase is involved in the acrosome reaction in sperm of the mussel mytilusedulis. *Mol Reprod Dev.* **67,** 465-471 (2004).

10. Lambert CC. Signaling pathways in ascidian oocyte maturation: the roles of cAMP/Epac, intracellular calcium levels, and calmodulin kinase in regulating GVBD. *Mol Reprod Dev.* **78,** 726-733 (2011).

11. Lambert, C. C. Signaling pathways in ascidian oocyte maturation: effects of various inhibitors and activators on germinal vesicle breakdown. *Dev Growth Differ.* **47,** 265-272 (2005).

12. Robert, L. K., Lucio-Gough, L. M., Goode, C. A., Mckinney, K. & Lambert, C. C. Activation of follicle cell surface phospholipase by tyrosine kinase dependent pathway is an essential event in Ascidian fertilization. *Mol Reprod Dev.* **54,** 69-75 (1999).

13. Su, Y. Q., Xia, G. L., Byskov, A. G., Fu, G. D. & Yang, C. R. Protein kinase C and intracellular calcium are involved in follicle-stimulating hormone-mediated meiotic resumption of cumulus cell-enclosed porcine oocytes in hypoxanthine-supplemented medium. *Mol Reprod Dev.* **53,** 51-58 (1999).

Supplementary Table S2: Comparison of composition of culture medium used in our present study and referred report.

Composition of culture medium	Su et al. 1999[1]	In the present study
Basic medium 199	With	With
Pyruvate	0.23mM	-
Glutamin	2mM	-
Bovine serum albumin	3mg/ml	-
Penicillin	100IU/ml	-
Streptomycin	100IU/ml	-
Hypoxanthine	4mM	-
Sodium pyruvate	-	0.91mM
Polyvinyl Alcohol	-	1mg/ml
D-glucose	-	3.05 mM
Gentamincin	-	1μg/ml
L-cysteine	-	0.57mM
Follicle stimulating hormone	-	0.5μg/ml
Luteinizing hormone	-	0.5μg/ml
Epidermal growth factor	-	10μg/ml

1. Su, Y. Q., Xia, G. L., Byskov, A. G., Fu, G. D. & Yang, C. R. Protein kinase C and intracellular calcium are involved in follicle-stimulating hormone-mediated meiotic resumption of cumulus cell-enclosed porcine oocytes in hypoxanthine-supplemented medium. *Mol Reprod Dev.* **53,** 51-58 (1999).

Supplementary Table S3: Primers used for real-time PCR.

Genes	Gene Bank accession No.	Primer sequence (5'-3')	Tm (℃)	Product size (bp)
Kdm6a	XM_003360275.3	F: GCAGGCTCAGTTGTGTAACC<br>R: GGTTTACATGCCTGCTGTGC	60	150
Setd2	XM_005669478.1	F: TAGCTCGCAAGCTGACTCAT<br>R: ACAGCCCCAAACTTCTGCAT	60	139
Ezh2	NM_001244309.1	F: TGCAACACCCAATACTTACAAGC<br>R: ACTCTTTTGCTCCCTCCAAGT	60	101
G9a	NM_001101823.1	F: GGAGGAGCTGGGGTTTGAC<br>R: CAGAGGTGGCTGCTGAGTTG	60	254
Eed	XM_013979231.1	F: TGTGACTATTCTTGGGCGATTT<br>R: TGGCTTTATGAGGATCTTCTACTT	60	153
Suz12	XM_013981159.1	F: TCAGGATATTCATCGCCAACC<br>R: CTTCGGATTCAAGAAATTCAGACA	60	134
Ywhag	XM_003124396.4	F: GGCCATGAAGAACGTGACAG<br>R: CATCCTGACATACGGCCTCC	60	224
Caspase3	XM_005671704	F: TTTGCGTGCTTCTAAGCCAT<br>R: GGCAGGCCTGAATTATGAAA	60	147
Bcl2	AF216205	F: CGTCCCAGCTCCACATCACC<br>R: AGTGCCCCACCGAAGGAGAA	60	130
Bax	XM_003127290.4	F: GCCGAAATGTTTGCTGACG<br>R: GCAGCCGATCTCGAAGGA	60	157

Supplementary Figures

Supplementary Figure S1: Effects of DMBA exposure on cumulus expansion of COCs. COCs were photographed using digital camera and the ratio of cumulus cell expansion was calculated according to the previously described method (Li et al., 2016). (**a**) Representative images of COCs taken at 0, 24 and 44h. Scale bar, 500μm. (**b**) The extent of the relative cumulus expansion of COCs presented as a ratio of the total two-dimensional area of each COCs at different time points to the original size of COCs at 0h. *** indicate significant differences at $P < 0.001$ level between groups.

Supplementary Figure S2: The effect of CBX on PB1 rate of porcine CDOs. PB1 rate of CDOs was not affected by 50μM CBX incubation for 44h.

Supplementary Figure S3: Full-length blot to detect p-ERK1/2, γ H2A.X and β-tubulin proteins. Lysate from three replicates of control and 20μM treated pig oocytes (see Methods for details) was run on the same gel and protein was transferred onto membrane. The membrane was cut in two parts containing the proteins above and below the 25 kDa marker. The upper part of the membrane was used to detect p-ERK1/2 (phospho T202/Y204) and the lower part was used to detect γH2A.X (phospho S139). After p-ERK1/2 detection, the upper part of the membrane was re-probed with anti-β-tubulin antibody.

Supplementary Figure S4: Distribution of nuclear stages of porcine oocytes from COCs treated by DMBA during IVM. (**a**) Representative images of porcine oocytes at different nuclear stages during IVM. Scale bar, 100μm. (**b-h**) Nuclear stages of porcine oocytes from COCs collected at 12h, 18h, 24h, 30h, 36h, 44, 72h, respectively. * indicates the significant difference at 0.05 level. * indicates significant differences at

$P < 0.05$ level between groups.

Supplementary Figure S5: Analysis of 1-cell arrested parthenotes from different groups. (**a**) Developmentally arrested 1-cell embryos were stained with Hoechst33342 to show different numbers of pro-nuclei, from 1 to 6. Scale bar, 100μm. (**b**) Graph to show the rate of 1-cell arrested parthenotes in control and 20μM DMBA treated COC and CDO groups. (**c**) Graph to show the rate of 1-cell arrested parthenotes with different number of pronuclei. *** indicate significant differences at $P < 0.001$ level between groups.

Supplementary Figure S1

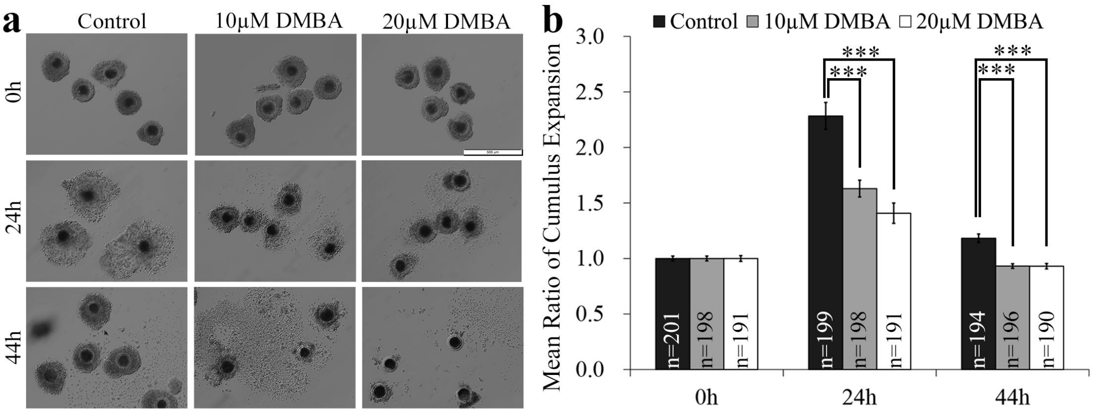

Supplementary Figure S2

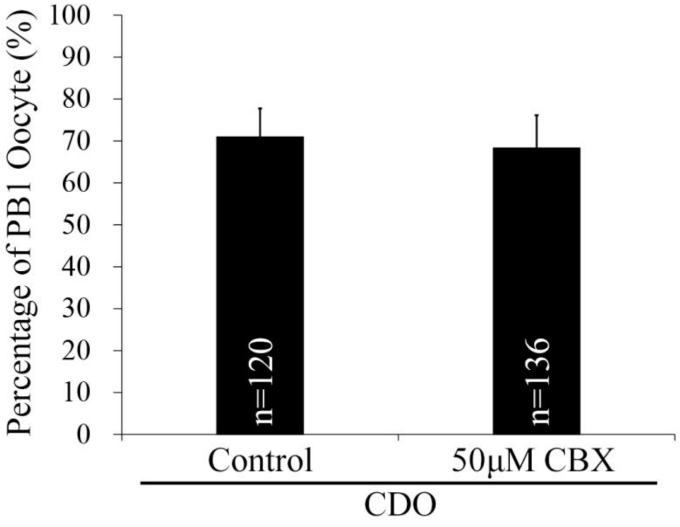

Supplementary Figure S3

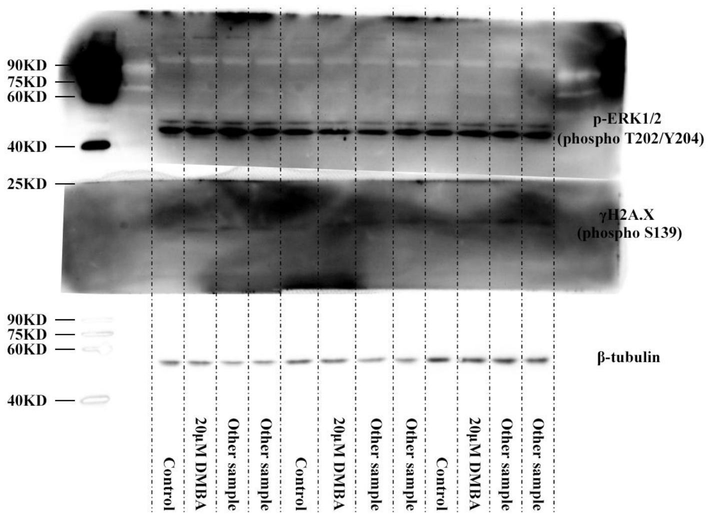

Supplementary Figure S4

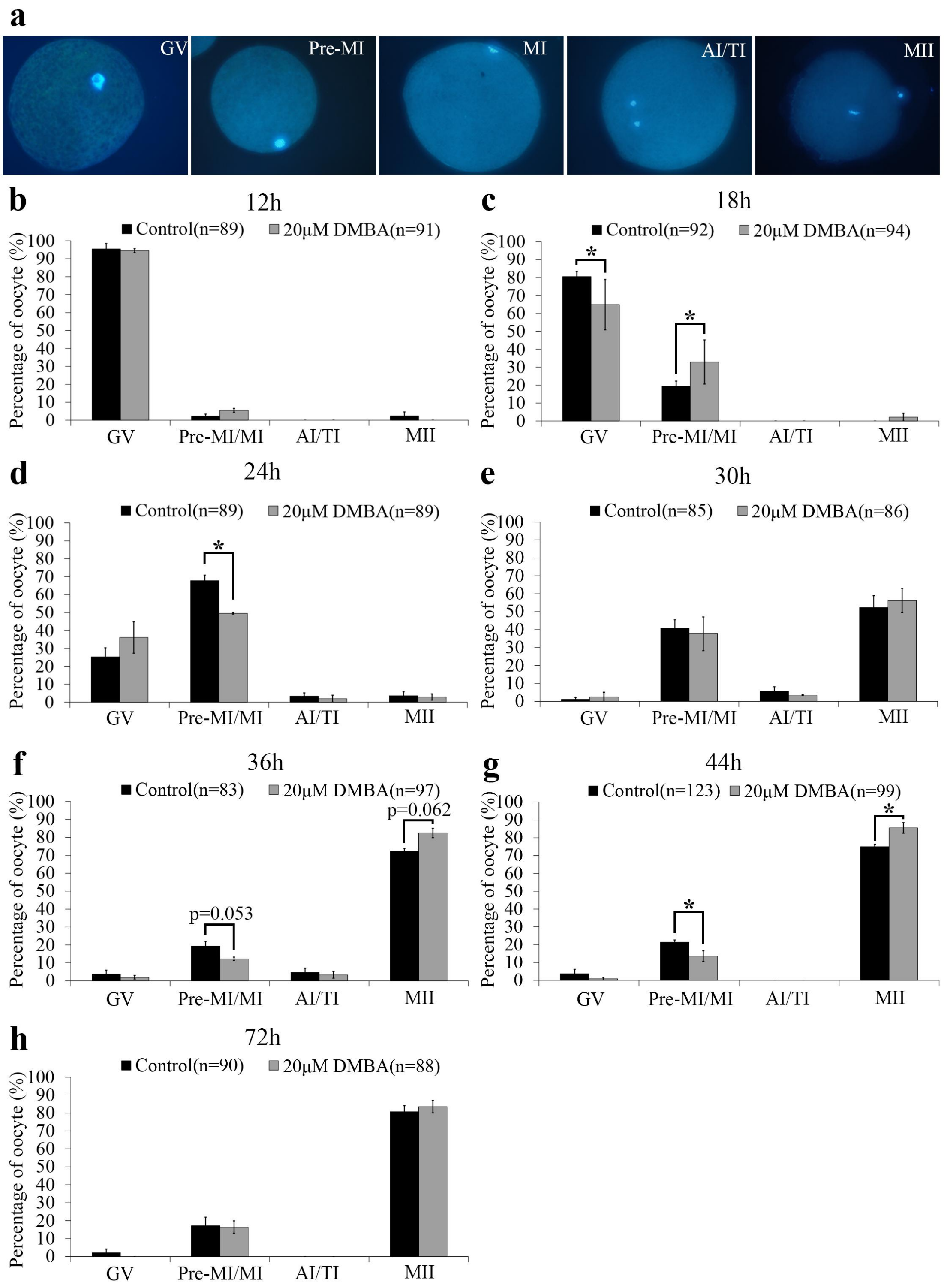

Supplementary Figure S5

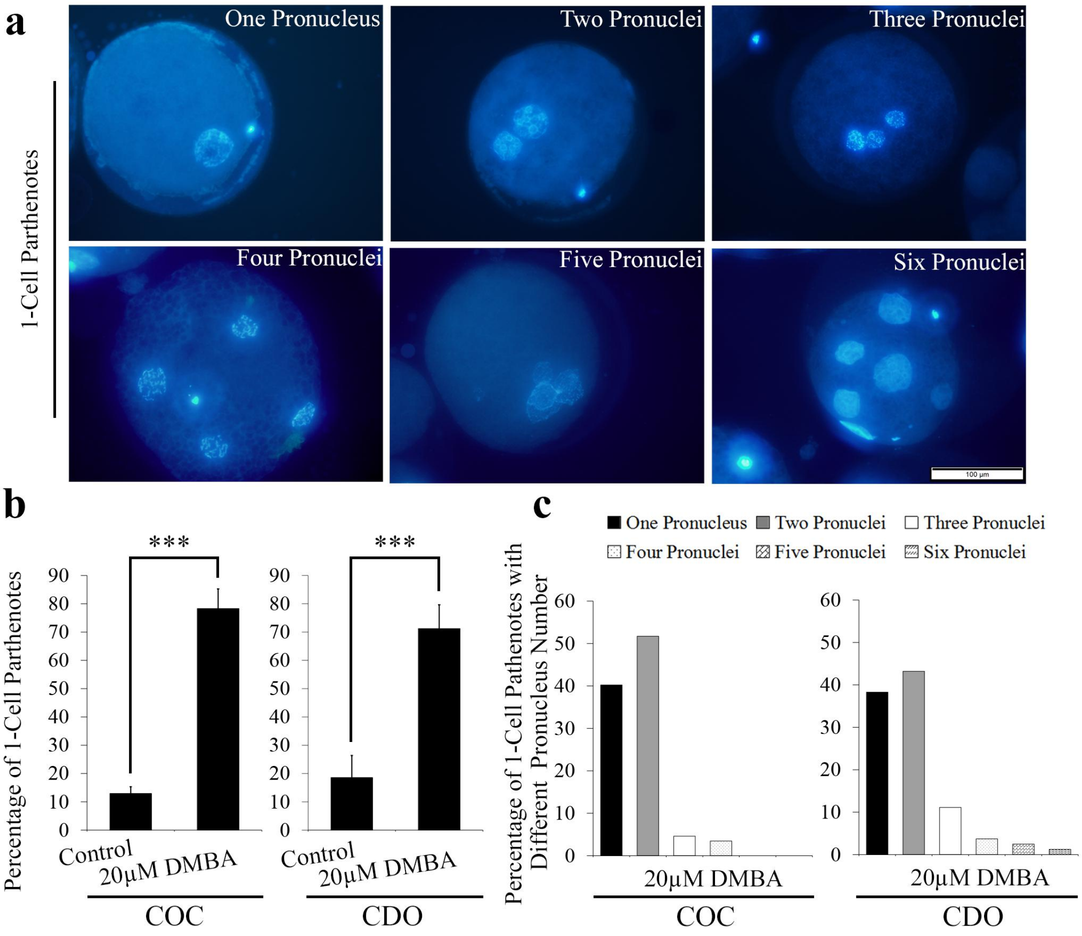

Supplement: Supplementary file 1 — Supplementary information [file 41598_2017_1870_MOESM1_ESM.pdf]
